# Social networks based on frequency of roost cohabitation do not reflect association rates of *Myotis lucifugus* within their roosts

**DOI:** 10.1002/ece3.7244

**Published:** 2021-05-02

**Authors:** Austin G. Waag, John J. Treanor, Jess N. Kropczynski, Joseph S. Johnson

**Affiliations:** ^1^ Department of Biological Sciences Ohio University Athens OH USA; ^2^ United States National Park Service Yellowstone National Park Mammoth Hot Springs WY USA; ^3^ School of Information Technology The University of Cincinnati Cincinnati OH USA

**Keywords:** association index, high‐frequency RFID, little brown bat, passive integrated transponder, radio‐frequency identification, social network analysis, Yellowstone National Park

## Abstract

Bats are a group of mammals well known for forming dynamic social groups. Studies of bat social structures are often based upon the frequency at which bats occupy the same roosts because observing bats directly is not always possible. However, it is not always clear how closely bats occupying the same roost associate with each other, obscuring whether associations result from social relationships or factors such as shared preferences for roosts. Our goal was to determine if bats cohabitating buildings were also found together inside roosts by using anti‐collision technology for PIT tags, which enables simultaneous detection of multiple tags. We PIT‐tagged 293 female little brown myotis (*Myotis lucifugus*) and installed antennas within two buildings used as maternity roosts in Yellowstone National Park. Antennas were positioned at roost entryways to generate cohabitation networks and along regions of attic ceilings in each building to generate intraroost networks based on proximity of bats to each other. We found that intraroost and cohabitation networks of buildings were significantly correlated, with the same bats tending to be linked in both networks, but that bats cohabitating the same building often roosted apart, leading to differing assessments of social structure. Cohabitation rates implied that bats associate with a greater number of their roost‐mates than was supported by observations within the roost. This caused social networks built upon roost cohabitation rates to be denser, smaller in diameter, and contain nodes with higher average degree centrality. These results show that roost cohabitation does not reflect preference for roost‐mates in little brown myotis, as is often inferred from similar studies, and that social network analyses based on cohabitation may provide misleading results.

## Graphical abstract

Sociograms depicting connections among little brown myotis within a maternity colony in Yellowstone National Park. Social networks based upon the frequency at which pairs of bats cohabitate the same roost (Panels a and c) connect bats that rarely or never were found roosting along in the same location on attic ceilings (Panels b and d). This comparison shows that co‐roosting associations in little brown myotis do not reflect bats' preference for specific roost‐mates, as is often interpreted in similar studies.

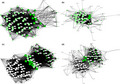

## 1 INTRODUCTION

Many species of bats gain benefits by forming social groups composed primarily of females during the breeding season. These assemblages reduce energy spent for thermoregulation (Willis & Brigham, 2007), provide opportunities for cooperative behaviors such as social grooming (Carter & Leffer, 2015; Kerth et al., 2003), and facilitate the transfer of information, such as the availability of alternative roosts (Kerth & Reckardt, 2003). The latter highlights that in many bat species, social groups use several roosts spread across the local landscape (Willis & Brigham, 2004). Bats within these groups, or colonies, asynchronously switch their roosting location frequently, creating fission–fusion social dynamics (Kerth & König, 1999). Fission–fusion dynamics serve as modulators for social intercourse (Aureli & Schino, 2019) in bat colonies because when any two bats switch roosts they become temporarily separated and cannot interact. Bats within species or populations that switch roosts frequently face variation in their roost‐mates each day, which may present cognitive challenges to maintaining relationships (Ramos‐Fernandez et al., 2018). Nevertheless, long‐term relationships do occur in some bat species despite frequent roost switching (Kerth et al., 2011) making bats an interesting group for studies of social ecology (Kerth, 2008).

Interactions between roost‐mates may reinforce relationships where fission–fusion dynamics exist. Notable examples of social interactions in bats include allogrooming in Bechstein's bats (*Myotis bechsteinii*) (Kerth et al., 2003) and food sharing in common vampire bats (*Desmodus rotundus*) (Carter & Wilkinson, 2013). However, social relationships between pairs of bats, or dyads, are often inferred based upon how many days bats spend together in the same roost (hereafter, roost cohabitation) because many bats roost in areas where their interactions are hard to observe without disturbance. Frequency of roost cohabitation is typically presented as an association index where greater values represent more days roosting together and stronger relationships. These relationships can be stable throughout the maternity season (Garroway & Broders, 2007) and across years (Zeus et al., 2018), suggesting a preference for specific roost‐mates (Kerth et al., 2011). Social network analyses based upon roost cohabitation, which we refer to as cohabitation networks, have revealed many aspects of social ecology in bats, including social structures based upon age (Patriquin et al., 2010), relatedness (Wilkinson et al., 2019), and breeding status (Zeus et al., 2018). Properties of cohabitation networks such as the number of ties (also known as edges, or links between animals in a network created by relationships) or the presence of subgroups have biological implications for members of the colony. For example, a high degree of connectivity among bats can influence the speed at which information or disease spreads among roost‐mates whereas these commodities might move slower through fragmented colonies of socially disconnected subgroups (Fortuna et al., 2009; Webber et al., 2016). Patterns of relationships also tell us how the environment influences social networks, as limited roost availability can lead to less diffuse networks where bats may be vulnerable to habitat loss or disturbance (Johnson et al., 2012).

**FIGURE 1:**
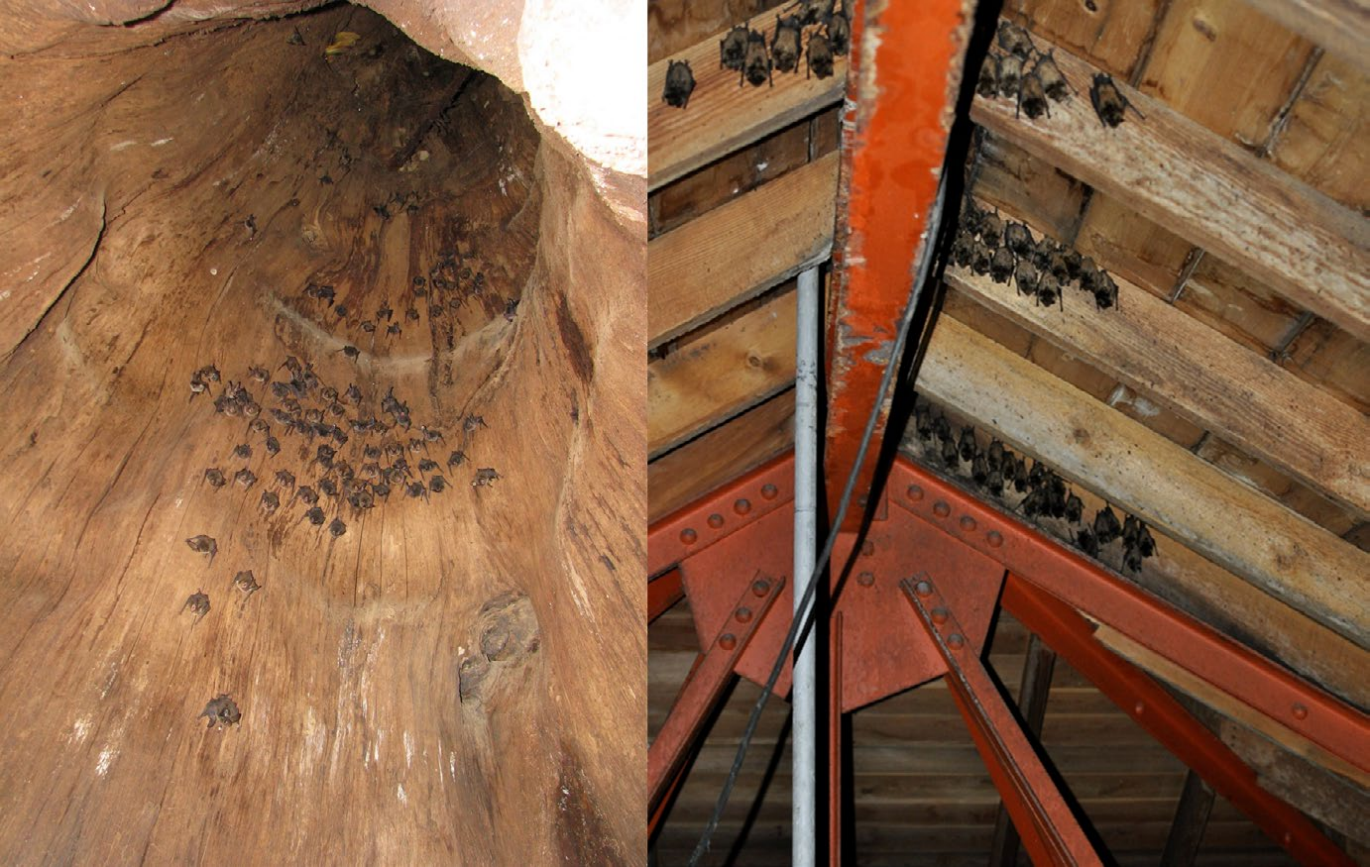
Rafinesque's big‐eared bats roosting in large tree hollows (left) and little brown myotis in building attics (right) do not necessarily associate in their roosts. Although network analyses describing social groups in bats often rely on frequency of roost cohabitation as the basis for relationships among bats, large social groups may develop subgroups within the roost or roost at random with other individuals, making it difficult to interpret the meaning of cohabitation networks alone

Drawing conclusions from relationships among members of a social group relies on accurately identifying those relationships. However, cohabitation relationships may be misleading if bats within the same roost do not encounter each other. This occurs in spacious environments such as tree hollows, caves, or buildings, where bats can be seen roosting close to some roost‐mates and far from others (Figure 1). Indeed, Willis and Brigham (2004) suggested that caves and buildings are analogous to forested areas where social groups inhabit multiple trees each day. If so, association indices based upon roost cohabitation would not accurately reflect preference for roost‐mates. Social networks emerging from cohabitation associations would still be meaningful in this scenario but would obscure patterns such as which bats tend to cluster together during the day (hereafter, intraroost networks). Dyads spending more time together likely have more interactions than those in different areas of the roost; therefore, intraroost networks may provide a more accurate indication of roost‐mate preference or the stability of relationships over time. Furthermore, comparing intraroost and cohabitation networks sheds light on what it means for bats to share a roost. For example, determining that most dyads with strong roost cohabitation associations have weak intraroost associations would suggest that shared roost choice explains more variation in cohabitation networks than preference for roost‐mates. This knowledge is important for conservation (Rhodes et al., 2006) and would not preclude some dyads having long‐term social relationships. Instead, it would indicate that these inferences could only be drawn from intraroost associations. Conversely, if dyads have strong relationships in both networks, then roost cohabitation can be used as a measure of social relationships, as it is often assumed to be.

The little brown myotis (*Myotis lucifugus*) is a North American bat species known to form large social groups in buildings, with individuals displaying high fidelity to roosts over relatively long life spans (Frick et al., 2010; Keen & Hitchcock, 1980). In buildings, trees, and bat houses, little brown myotis switch roosts throughout the maternity season (Slough & Jung, 2020), resulting in fission–fusion dynamics. In trees, this species switches roosts every 1–6 days (Olson & Barclay, 2013) although groups inhabiting more permanent structures such as buildings may move less frequently (Lewis, 1995). It is unknown if little brown myotis develop preference for roost‐mates within these colonies, but their ability to recognize echolocation calls of individual conspecifics (Kazial et al., 2008) indicates they may have the cognitive ability to do so. However, earlier studies of the vocal repertoire (Barclay et al., 1979) and mating behaviors (Barclay et al., 1979) of little brown myotis suggest a lack of complex social interactions and revealed that social groups form primarily for thermoregulatory purposes. Thus, the little brown myotis is an ideal species for comparisons of cohabitation and intraroost networks because colonies inhabiting buildings are likely to cohabitate roosts, but these relationships may not be representative of associations within the roost.

Passive integrated transponder (PIT) tags are frequently used to create cohabitation networks (Garroway & Broders, 2007; Kerth & König, 1999; Patriquin et al., 2010) and may be leveraged to generate intraroost networks as well. Weighing 0.1 g, PIT tags do not contain internal batteries; instead, they receive power from radio waves emitted from antennas operating on the same frequency. PIT tag readers autonomously and continuously record the unique identity of tags when they come within range of antennas, making them seem ideal for passive studies of bats at known roosts (van Harten et al., 2019). However, PIT tags used in wildlife research employ low‐frequency (125–135 kHz) technology, which is hampered by a radio‐frequency identification (RFID) limitation known as tag collision. Tag collision occurs when >1 tag is present at an antenna and results in readers failing to detect tags due to a blockage of transmissions (Klair et al., 2010; Shih et al., 2006). The effects of tag collision are well recognized in the fields of RFID (Klair et al., 2010; Shih et al., 2006) and animal agriculture (Adrion et al., 2018; Reiners et al., 2009; Thurner et al., 2009), but are rarely discussed in wildlife studies despite the limitations they create (Smyth & Nebel, 2013). For example, tag collision limits the placement of PIT tag antennas to movement paths when studying PIT‐tagged bats at their roosts (Garroway & Broders, 2007; Kerth & König, 1999; O’Shea et al., 2011). Tag collision can be avoided by using high‐frequency (13.56 MHz) readers and antennas with inherent anti‐collision capabilities (Klair et al., 2010). High‐frequency readers also have increased data transfer rates and tag read speeds, allowing readers to record up to 100 tags simultaneously, and record tags four times per second at a single antenna (Klair et al., 2010). Using high‐frequency readers with anti‐collision technology therefore permits antennas to be placed within areas where animals collectively congregate or move quickly through the range of antennas.

The goal of our study was to determine if dyads commonly found cohabiting buildings were also found together inside roosts. We hypothesized that bats in cohabitation networks would have more ties than bats in intraroost networks because buildings are spacious environments that provide bats with opportunities to interact with some roost‐mates more than others. We predicted that cohabitation and intraroost networks would be correlated to each other, meaning that many of the dyadic relationships among bats would be consistent in the two networks, but that cohabitation networks would have more connected dyads, fewer intermediaries between unconnected bats, and consist of nodes (bats) with more connections to other bats than intraroost networks. These network measures describe the tendency towards subgroupings and how quickly information or disease can spread through a group. By comparing these measures between networks, we illustrate how knowledge of roost associations can improve understanding of social structure and ecology of bat populations.

## 2 MATERIAL AND METHODS

### 2.1 Data collection

Our study was conducted in Lamar Valley and Mammoth, Wyoming, on the northern range of Yellowstone National Park (44.9769°N, 110.6991°W). Previous work in these areas found female little brown myotis to roost primarily in buildings and that bats in the Lamar Valley do not roost with bats in Mammoth, 37 km to the west (Johnson et al., 2017, 2019). From 2015 to 2018, we conducted a single night of bat capture outside a maternity roost in each area during late July, after the majority of bats have given birth. We captured little brown myotis with mist nets and subcutaneously implanted females with 12 mm high‐frequency PIT tags (HID Global). High‐frequency readers (HDX Multi‐antenna Reader, Oregon RFID), and high‐frequency antennas (FEIG Electronic) were installed inside the attics of one building in Lamar Valley and one in Mammoth. Bats roost within the attics of these buildings, with the attic in Mammoth measuring 288 m^2^ and the attic in Lamar measuring 195 m^2^. These roosts are used by several hundred bats each year. At Lamar Valley, this included 62 tagged bats in 2017 and 85 in 2018. At Mammoth, this included 56 tagged bats in 2018. Although we cannot estimate what proportion of each colony was tagged during each year, it is likely that we did not tag the majority of bats in either group. Readers continuously recorded the date, time, tag identification number, and specific antenna that made the scan.

**FIGURE 2:**
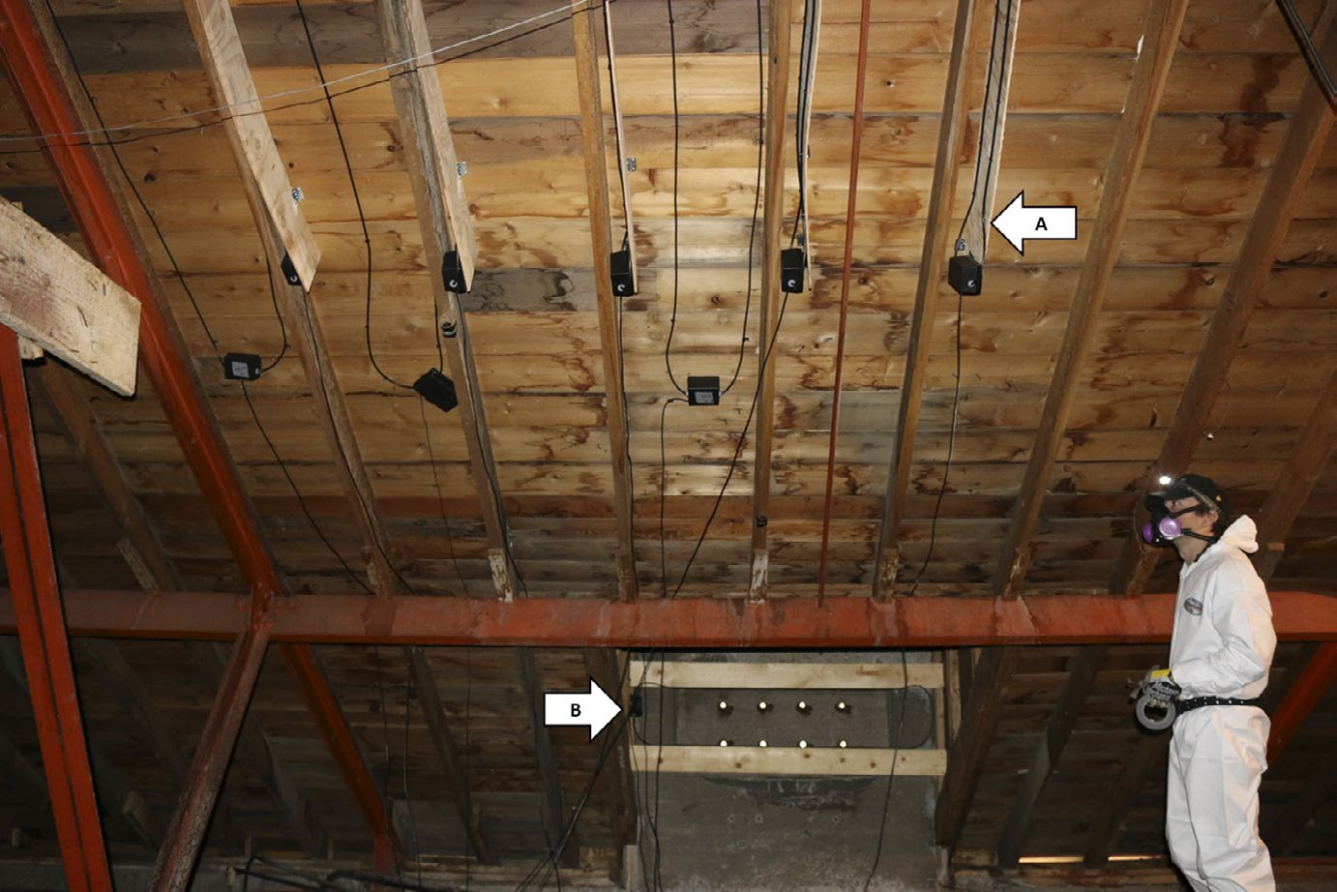
PIT tag antennas placed along an attic ceiling (arrow “A”) and entryway antennas that bats traverse through to access the roost (arrow “B”), inside a little brown myotis maternity roost in Yellowstone National Park, USA. Ceiling antennas were placed at two different orientations. Antennas were positioned perpendicular to the attic ceiling on plywood segments and flush to the attic ceiling to increase tag readability through different orientations of antennas relative to PIT‐tagged bats. Read ranges of antennas did not overlap

Antennas were constructed of a motherboard and RG6 18AWG coaxial cable, shaped into a 1.5 × 0.4 m or 1 × 0.2 m rectangle. Two antennas, which were constructed and tested to possess a read range of 20 cm, were installed over entrances at both roosts (Figure 2). The read range of these antennas entirely covered primary attic entryways, where an estimated >95% of the colony entered and exited each roost (Waag, 2018). An additional eight antennas at Lamar Valley and 16 antennas at Mammoth were positioned on attic ceilings in areas where bat activity such as urine and guano staining were visible (Figure 2). Ceiling antennas were attached vertically to the attic roof, in addition to being installed flush to the ceiling to maximize the surface area where bats could be detected. Antenna segments were scarred to encourage bats to roost on them. Antennas were tested by detecting multiple tags simultaneously for several weeks to confirm that tag‐collision was not occurring. We installed antennas and readers during spring of 2016, before colonies formed in May, but logistical constraints and equipment prevented data collection until 2017 at Lamar Valley and 2018 at Mammoth.

### 2.2 Data analysis

We constructed six social networks, consisting of three pairs of intraroost and cohabitation networks during the maternity season (10 June–15 August) in 2017 and 2018: Lamar Valley 2017, Lamar Valley 2018, and Mammoth 2018. Data from different years were kept separate because the composition of the network is expected to change between years due to births, deaths, dispersal, and addition of tagged bats. We chose to construct these networks based upon the entire maternity season because our goal was to use intraroost networks to evaluate cohabitation networks, which are typically based on seasons (Bachorec et al., 2020; Chaverri, 2010) or years (Kerth et al., 2011; Wilkinson et al., 2019). Data collected from Mammoth readers in 2017 were not included because antennas were placed in areas within the roost that few bats visited, and antennas were relocated in 2018.

Networks were created in UCINET (Analytic Technologies) (Borgatti et al., 2002). Cohabitation networks were created from one mode (bat‐by‐bat) matrices weighted based upon the number of days each dyad was detected using roost entryways during the same day (i.e., frequency of cohabitation). Intraroost networks were also created using one mode matrices but were weighted based on the amount of time bats spent roosting together at the same antenna. Because antennas scanned tags four times per second, we often recorded several bats simultaneously, followed by seconds or fractions of a second with a different combination of tags or no tags at all. We considered it unlikely that bats were moving throughout the roost this rapidly, and that tags were sometimes missed because small movements could cause a tag to briefly be outside of its read range. Thus, we collapsed data into more meaningful time periods before creating weights. Tags detected at the same antenna were partitioned into 5‐min periods to determine which bats roosted together. Weights in intraroost networks were therefore based upon the amount of time, rounded into 5‐min bins, that dyads were documented at the same location within the roost, while cohabitation networks were based solely on the frequency that bats were found in the same roost. Any bat detected at least once was considered part of the network.

We calculated the degree of correlation for each pair of networks using a quadratic assignment procedure (QAP) regression in UCINET to determine how often dyads found cohabitating roosts were found together within the roost. This analysis expresses the amount of similarity between dichotomous networks (with weights removed) as a percent. The likelihood that the observed similarity in the two networks could be observed by chance is given as a *p*‐value based on 10,000 permutations of the observed one mode matrices (i.e., the whole network). We performed this analysis with weights removed because the objective was to determine the extent that ties created through cohabitation reflected ties created by roosting together. We did not analyze association strengths using the QAP procedure because strengths had lower maximum values in cohabitation networks because they were based on days compared to associations in intraroost networks, which were based on time. In order to compare relationship strength, we created heat maps based on dyadic associations in each network. The strength of associations is visualized with warmer colors and allows for visual comparisons of associations between networks.

To compare the properties of cohabitation and intraroost networks, we calculated the following measures for each network in UCINET: average degree centrality, whole network centralization, network density, and network diameter. Average degree centrality is the average number of ties each bat has to others, either based on roost cohabitation or being detected at the same antenna together. The difference in average degree centrality between networks represents the average number of bats each individual is connected to in one network, but not the other. Whole network centralization is a measure of how centered a network is on a few bats. In both types of networks, greater centralization reflects social groups where a single or a few bats have a disproportionately larger number of connections compared to others in the social group. Network density is the proportion of bats sharing a tie in a network, which is determined by dividing the number of observed ties by the total number of ties possible. A relatively dense cohabitation network would be one where most of the social group roosts in the same structure on at least one occasion, while less dense networks would indicate a larger number of bats are isolated. Comparatively, a dense intraroost network would be one where many bats are found together within the roost and a less dense network would be one where bats were uncommonly observed together. Finally, network diameter is the longest geodesic distance between two bats. In both networks, small diameters represent social groups where dyads not tied together are separated by fewer intermediaries, similar to the cultural idea of “six degrees of separation”.

## 3 RESULTS

**FIGURE 3:**
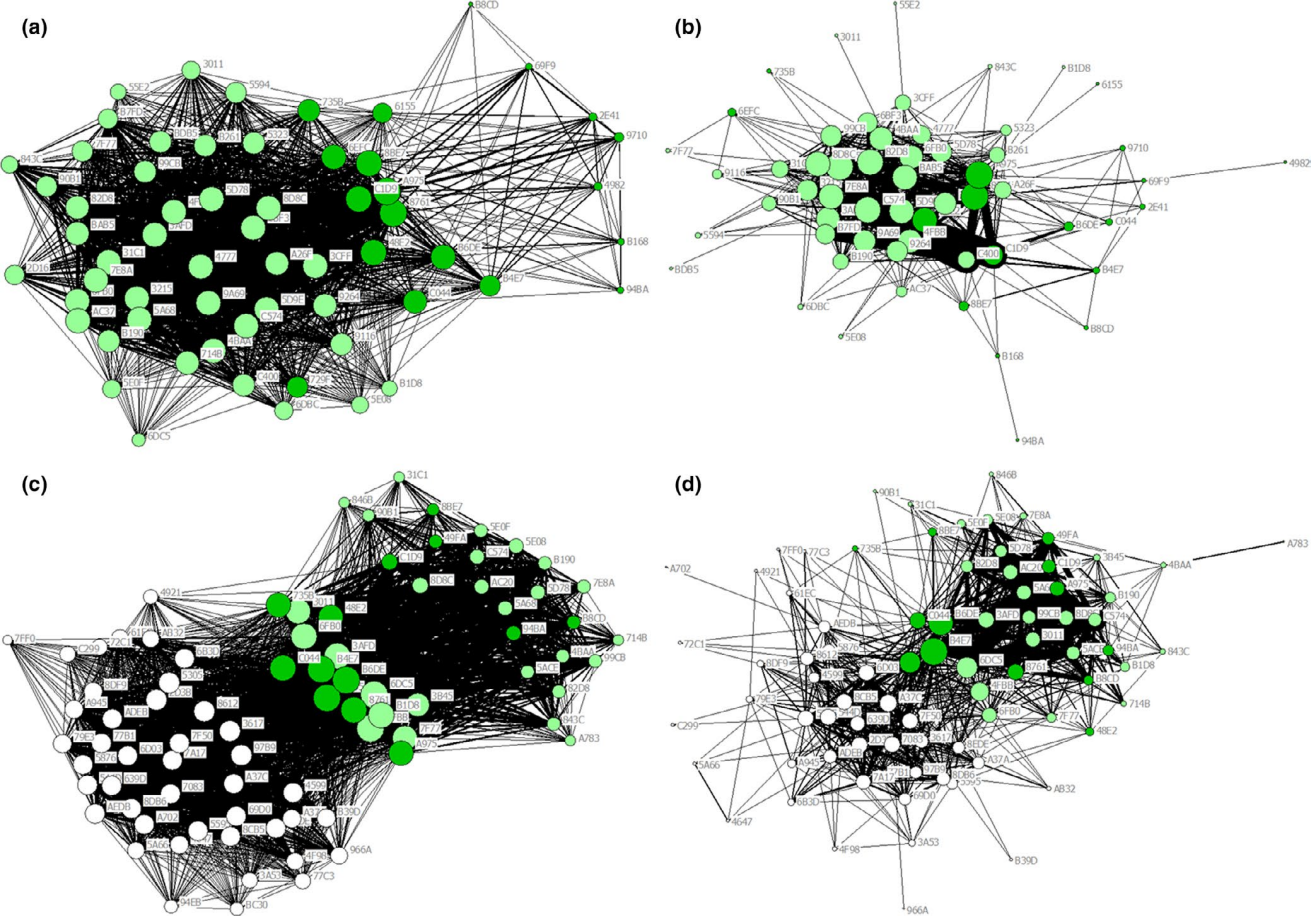
Sociograms of the Lamar Valley little brown myotis maternity colony based on cohabitation association rates (Panels a and c) and intraroost association rates (Panels b and d). Cohabitation networks were denser, smaller in diameter, and composed of nodes with higher degree centrality than intraroost networks. Nodes represent bats tagged with high‐frequency PIT tags during the summers of 2015 & 2016 (dark green), 2017 (light green), and 2018 (white). Bats never detected roosting with another tagged bat are not shown. Node size reflects degree centrality (larger nodes have more ties to other bats in the colony) and tie width represents the strength of ties between any two bats. Panels a and b were generated from data collected between June and August 2017, and Panels c and d were based upon data collected between June and August 2018

**FIGURE 4:**
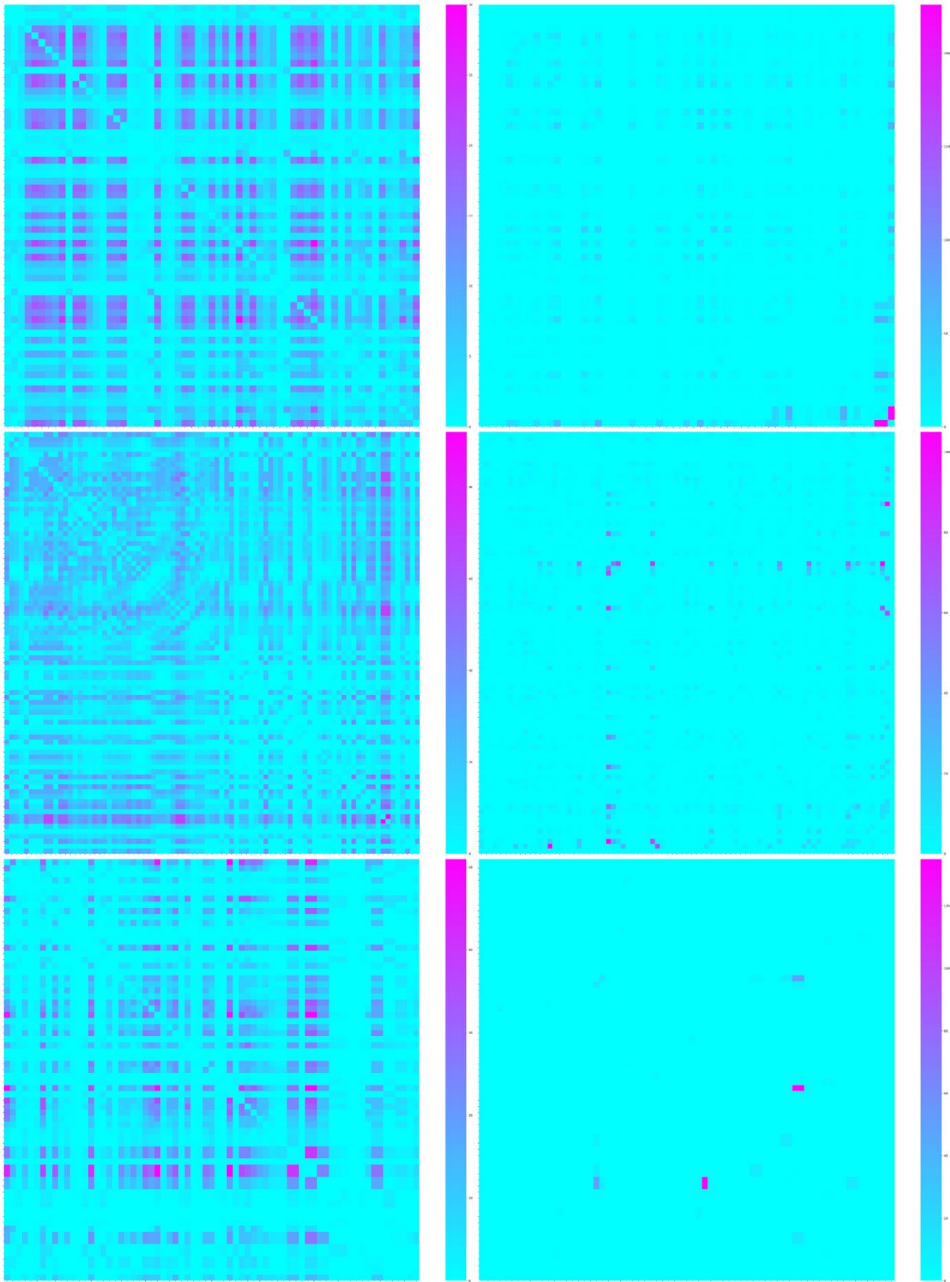
Heatmaps displaying dyadic association rates for little brown myotis based on cohabitation frequency (left column) did not resemble associations based on locations of bats within the roost (right column). Data collected from Lamar Valley during 2017 are shown in the top two maps, data collected for this roost during 2018 are shown in the center two, and data collected from Mammoth during 2018 are shown along the bottom. Warmer colors denote stronger associations in each heat map. For each pair of networks (cohabitation and roosting), rows and columns represent the same bats in the same order, and the diagonal is omitted because it would represent a bat's association with itself. Association strength for any given dyad in the cohabitation network can be compared directly to the same cell in the intraroost network to the right

We tagged 293 female little brown myotis with PIT tags between 2015 and 2018. We found that cohabitation and intraroost networks tied many of the same dyads together, but relationships based on cohabitation over‐estimated the number and strength of connections. At Lamar Valley, cohabitation and intraroost networks were significantly correlated during 2017 (*p* < 0.001, Figure 3a,b) and 2018 (*p* < 0.001, Figure 3c,d) but were only 58% similar in each year. This means that although dyads tied together in one network were significantly more likely to be tied together in the other network than expected by chance alone, there were many bats tied together by cohabitation that were not detected together within the roost. Specifically, while >60% of all possible ties were formed in the cohabitation networks, <30% of all possible ties were formed in intraroost networks (Table 1). On average, individual bats in Lamar Valley had more than twice the number of ties when relationships were based on cohabitation (53 in 2017 and 45 in 2018) than when relationships were based on clustering within the roost (20 in 2017 and 17 in 2018). Heat maps of association strength showed little correspondence between the cohabitation and intraroost networks. Many dyads linked with strong ties in cohabitation networks lacked or had relatively weak ties in intraroost networks (Figure 4). Furthermore, many of the strongest ties in intraroost networks were not strong ties in cohabitation networks.

**TABLE 1:** Network statistics for two maternity colonies of little brown myotis in Yellowstone National Park, June to August 2017 and 2018

Roost	Network	Year	Nodes	Density	Diameter	Network centralization	Degree centrality
Lamar Valley	Cohabitation	2017	61	0.74	2	0.27	44.6
Lamar Valley	Intraroost	2017	57	0.31	5	0.33	17.1
Lamar Valley	Cohabitation	2018	83	0.64	2	0.48	52.8
Lamar Valley	Intraroost	2018	81	0.25	5	0.36	20.3
Mammoth	Cohabitation	2018	56	0.56	2	0.10	31.1
Mammoth	Intraroost	2018	51	0.08	9	0.43	2.3

Note

Cohabitation network measures were constructed and weighted upon the frequency at which dyads occupied the same roost. Intraroost network measures were constructed and weighted based upon the amount of time bats spent at the same location within the roost.

**FIGURE 5:**
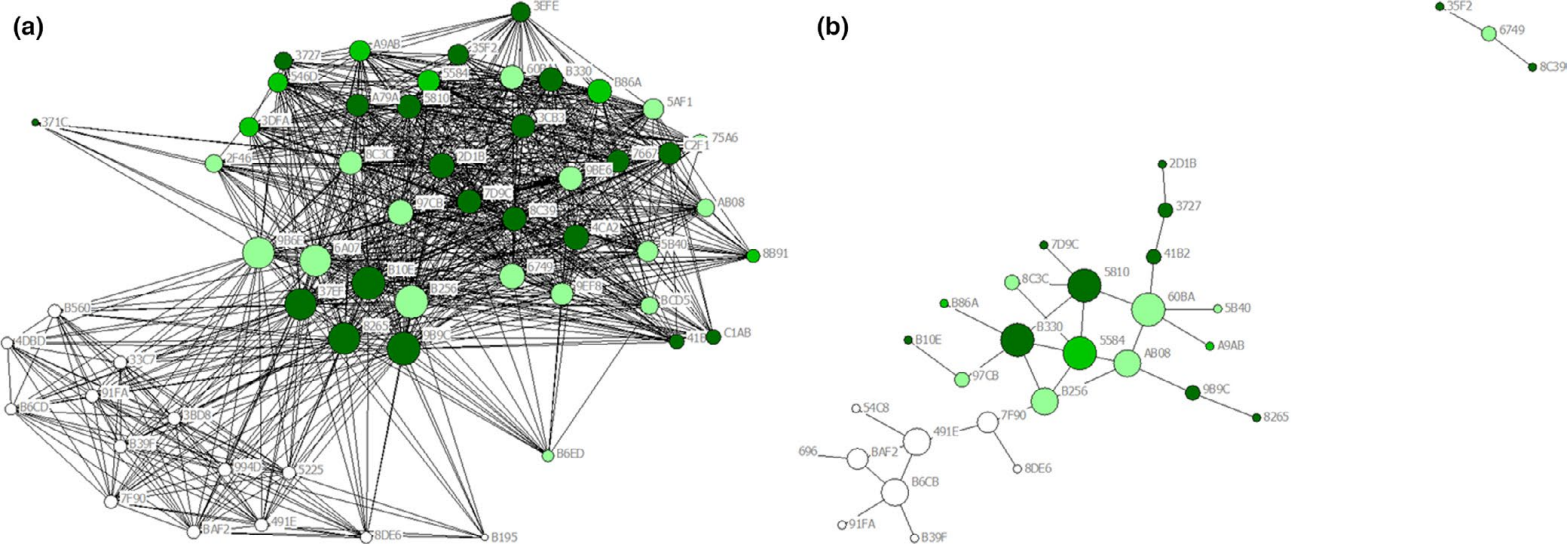
Sociograms of the Mammoth little brown myotis maternity colony based on cohabitation association rates (Panel a) and intraroost association rates (Panel b). The cohabitation network was denser, smaller in diameter, and composed of nodes with higher degree centrality than the intraroost network. Nodes represent bats tagged with high‐frequency PIT tags during the summers of 2015 & 2016 (dark green), 2017 (light green), and 2018 (white). Bats never detected roosting with another tagged bat are not shown. Node size reflects degree centrality (larger nodes have more ties to other bats in the colony) and tie width represents the strength of ties between any two bats. Panels a and b were generated from data collected between June and August 2018

Cohabitation and intraroost networks were also significantly correlated at Mammoth (*p* < 0.001) but were only 15% similar. Thus, although bats were more likely to be linked in both networks than expected by chance, most dyads connected in the cohabitation network were not found roosting together (Figure 5). Less than 10% of ties were formed in the intraroost network compared to nearly 60% of ties based on cohabitation. On average, bats at Mammoth had more than 10 times the number of ties when relationships were based on cohabitation (31 per bat) than intraroost clusters (two ties per bat). Many dyads with strong associations in the Mammoth cohabitation network had weak or no association in the intraroost network (Figure 4).

Intraroost networks were also larger in diameter at both buildings. While almost all bats were connected by single ties in cohabitation networks, nodes in intraroost networks were separated by as many as five ties at Lamar Valley and nine ties at Mammoth (Table 1). This is shown in the sociograms of cohabitation networks, where bats tagged during the summer were not always detected in the roost on at least one day with another member of the colony, resulting in a network diameter of two (Figure 3, Figure 5). Sociograms of intraroost networks show that bats sharing the roost on the same day were often not detected at the same antennas, creating larger diameter networks. This was most pronounced at Mammoth where many bats were detected roosting with only one, or often no, tagged roost‐mate (Figure 5b). Unlike the previous measures, network centralization was broadly similar between the two network types at Lamar Valley, meaning that neither network tended to be centered around a few bats. At Mammoth, the intraroost network was markedly more centralized than the cohabitation network, meaning that in the intraroost network, the average bat had fewer connections to others in the roost, creating a more spread‐out network.

## 4 DISCUSSION

We found that associations among little brown myotis based upon frequency of roost cohabitation result in networks that do not relate to associations within the roost itself, as bats that frequently roosted in the same building were rarely found in the same areas within the roost. Frequency of cohabitation is routinely used as a measure of the strength of social relationships in bats, either implicitly or explicitly assuming bats roosting together more interact more (Patriquin et al., 2010; Wilkinson et al., 2019). While this is likely true in many instances, our study shows this assumption is not valid for little brown myotis in spacious roosting environments such as buildings. Although dyads’ rates of roost cohabitation did not reflect their associations within roosts, this does not mean that roost cohabitation lacks importance. Instead, it suggests that relationships based on cohabitation are more representative of shared preference for roosts than for roost‐mates. Accurate interpretation of associations commonly used to create social networks in bats is important because failure to do so could lead to misleading conclusions about social ecology or environmental drivers of perceived social structures.

In their study of big brown bats (*Eptesicus fuscus*), Willis and Brigham (2004) suggested that availability of trees in forests provide bats with opportunities to interact with other members of the colony in the same way that spacious roosts, such as buildings do. Our study of little brown myotis in buildings provides some support for this hypothesis, as we found that bats often roosted apart within buildings and were spread throughout attics in a manner comparable to bats roosting in different trees (Fortuna et al., 2009; Garroway & Broders, 2007; Silvis et al., 2015). Although buildings provided little brown myotis with opportunities to roost with other colony members, only a small number of the total possible ties were formed in intraroost networks, indicating that subgroups may exist within buildings. Our finding that bats within the same attic on the same day often roost apart suggests that simulating spread of socially communicable commodities such as disease in building roosts may not be as straightforward as roost cohabitation suggests (Webber et al., 2016). Our results also speak to the conservation value of spacious roosts, such as buildings, which may provide bats with high quality habitat (Johnson et al., 2019) analogous to forests patches.

In our study, the assumption that roost cohabitation is synonymous with roosting together would have led to misleading conclusions about social structure. For example, intraroost and cohabitation networks had different values for important characteristics such as density. Density represents how closely individuals in a network are connected (Wasserman & Faust, 1994; Wey et al., 2008), and has been used to assess connectivity among communities in social bat species (Wilkinson et al., 2019). In our study, cohabitation networks had at least twice the amount of connectivity of intraroost networks, incorrectly implying a single, densely connected community with many strong ties. Similar networks were observed among big brown bats roosting in buildings and were used to determine that pathogens would spread more rapidly in buildings than among bats roosting in trees (Webber et al., 2016). Our results suggest that for little brown myotis, interpreting cohabitation networks in these ways may be misleading. We found that cohabitation also yielded an exaggerated sense of connection among bats in terms of average degree centrality. High average degree centrality of bats within cohabitation networks suggests that bats roosting in buildings encountered and formed relationships with most of the other bats in the roost when, in fact, these bats frequently roosted in different areas of the building attics.

Understanding the significance of associations among bats within a colony is important because comparisons of networks built upon these relationships are increasingly made within and among species. Among species, differences in network density may reflect variation in the likelihood that subgroups form within the larger network (Wilkinson et al., 2019). Within species, habitat availability can also influence social structure. For example, comparatively dense cohabitation networks of different populations of Rafinesque's big‐eared bats (*Corynorhinus rafinesquii*) were found to be associated with limited roost availability (Johnson et al., 2012). Network cohesion influences the rate at which information or disease can spread through the colony and can be interpreted through measures of network diameter. All three of the cohabitation networks in our study had a diameter of two, meaning that two links was the greatest social distance observed between any two bats in the colonies. Thus, information or diseases communicable from bat‐to‐bat contact is within only a single degree of separation from the entire colony at any point in the network. However, intraroost networks had diameters ranging from 5 at Lamar Valley to 9 at Mammoth, reflecting a more diffuse group where bat‐to‐bat transmission would likely be much slower. Little brown myotis roosting in maternity colonies are frequently infested with ectoparasites (Webber et al., 2015), and larger diameter networks may limit their spread. Conversely, little brown myotis benefit from knowledge of alternative roosts on the landscape and more diffuse networks may hinder the sharing of information about roost availability (Kerth & Reckardt, 2003). We emphasize that our measures of network diameter were taken at the scale of the entire maternity season, and studies interested in transmission of information or disease would need to base their findings on an appropriate scale. Regardless, future studies involving roost associations may miss important aspects of network structure if based upon roost cohabitation.

The two buildings we monitored differed in the area available to roosting bats. Bats at Mammoth roosted in an area 32% larger than the Lamar Valley roost, which may be why relatively few bats were tied in the intraroost network at Mammoth. Unlike the roost at Lamar Valley, signs of roosting bats were ubiquitous throughout the roost at Mammoth and bats were often observed roosting throughout the attic space where antennas could not always be placed. Thus, we may have missed instances of tagged bats roosting together that would have resulted in more ties, and our sampling methods would have benefited from a higher density of antennas at Mammoth. The Lamar Valley roost was smaller and roost locations required fewer antennas to effectively cover. We recommend future researchers consider difficulties in covering large areas with antennas when designing similar studies. Our methods would have also benefited from a larger sample of tagged bats. It is likely that many of the bats that were isolated (having no ties) in intraroost networks roosted with one or more of the dozens of unmarked bats. Similarly, the number of ties we observed for each bat in all networks is likely an underrepresentation due to the number of unmarked bats. We therefore urge caution when making inferences of social structure in little brown myotis from our results. However, we can still confidently conclude that bats in the same building often roosted apart because nearly the entire sample of tagged bats were located while roosting on the ceiling alone more often than with another tagged bat. At both roosts, the read range of our PIT tags was likely reduced due to the orientation of tagged bats relative to antennas (Klair et al., 2010). However, we were able to minimize this limitation of ceiling antennas through careful antenna design. We strongly recommend that similar care is taken to ensure read ranges sufficiently cover desired areas in future studies.

Our study found that social networks based solely on cohabitation of roosts do not reflect associations among little brown myotis within their roosts and therefore provide more insight into patterns of roost use than social relationships. Assuming that bats inhabiting the same roost associate with each other obscures interesting variation in the strength of ties that are important to social structure and may imply the presence of social relationships where they do not exist. In the case of little brown myotis roosting in buildings, focusing on roost cohabitation resulted in networks with dense ties and nearly no social distance within colonies even though tagged bats often roosted in different areas within building attics. These results highlight the need to look at associations within the roosts at time scales finer than daily cohabitation when studying social structures of bats.

## CONFLICT OF INTEREST

No competing interests.

## AUTHOR CONTRIBUTION

**Austin G. Waag:** Conceptualization (supporting); data curation (lead); formal analysis (supporting); funding acquisition (supporting); investigation (lead); methodology (equal); project administration (supporting); resources (supporting); software (lead); supervision (supporting); validation (equal); visualization (equal); writing – original draft (lead); writing – review and editing (lead). **John J. Treanor:** Conceptualization (supporting); data curation (supporting); formal analysis (supporting); funding acquisition (equal); investigation (supporting); methodology (equal); project administration (supporting); resources (equal); software (supporting); supervision (supporting); validation (supporting); visualization (supporting); writing – original draft (supporting); writing – review and editing (supporting). **Jess N. Kropczynski:** Conceptualization (equal); data curation (supporting); formal analysis (lead); funding acquisition (equal); investigation (supporting); methodology (equal); project administration (supporting); resources (supporting); software (supporting); supervision (supporting); validation (equal); visualization (equal); writing – original draft (supporting); writing – review and editing (supporting). **Joseph S. Johnson:** Conceptualization (equal); data curation (supporting); formal analysis (supporting); funding acquisition (equal); investigation (supporting); methodology (equal); project administration (lead); resources (equal); software (supporting); supervision (lead); validation (supporting); visualization (supporting); writing – original draft (supporting); writing – review and editing (supporting).

## Data Availability

Data used to create social networks are available on Dyad (https://doi.org/10.5061/dryad.rv15dv47b).
